# The Health Food Company

**Published:** 1876-10

**Authors:** 


[From *Monthly Record*, Brooklyn, October, 1876.]

### *THE HEALTH FOOD COMPANY.*

"What shall we eat?" has been the absorbing inquiry during the centuries, and never more so than in the present day, when nature is so prolific in supplying such an abundance of food for the use of man. It is somewhat strange that to our food may be attributed a large proportion of the physical ills that flesh is said to be heir to. The normal condition of man is *healthy*, and he would continue so if his animal nature was kept in abeyance or regulated by his intellectual. "Do thyself no harm," is as applicable to the physical constitution of man as to his moral nature. We are told, "that as the ultimate element of all organic bodies is nearly the same, one substance is as good as another for human food." But from the laws of constitution and relations, we may set it down that as a general statement the simpler, plainer, and more natural the food of man is, the more perfectly those laws are fulfilled, and the more healthy, vigorous, and long-lived will be the body, and the more perfect will be all the senses, and the more active and powerful the intellect and moral faculties. By simple food, we mean that which is not compounded and complicated by culinary processes, that which is not dressed with pungent stimulants, seasonings, or condiments ; but that which the Creator has assigned for man, and in such conditions as are best adapted to the human system.

These remarks are suggested by the following communication of our respected correspondent, the Rev. J. Bastow, Baptist minister, Port Chester, N. Y., to which we invite the special attention of our readers. Mr. B. speaks from *personal experience,* he having suffered a prolonged martyrdom from the affliction referred to, and incurred a vast amount of expense in medical treatment, prior to his being providentially directed to use the cereal preparations of the "Health Food Company," which he "*intelligently and heartily recommends.*', We also have many personal friends who are using these preparations *daily,* and who speak in the highest terms of their beneficial effect. EDITOR.

---

### NEW PREPARATIONS OF WHEAT.

We are accustomed justly to speak of health as the best of all temporal blessings, and no one realizes this better than those who have for years suffered for the want of it ; and especially from any of the numerous diseases of the mucus membrane. Long continued poor health, however, teaches us one thing very clearly, viz., that when a chronic disease is once fixed upon us, medicine can do very little to remove it. Even the doctors are more and more frankly confessing this among themselves and to their educated patients. Medicine has very great power to destroy, but very little power to restore, heal, and reconstruct. I am not saying this to disparage the medical profession. I count among them a large number of warm, personal friends, who are very useful, broad-minded large-hearted, and self-sacrificing. But the most candid and honest among them are turning their attention, ever increasingly, from medicine to careful nursing and a wise use of hygienic principles. They tell us, that after all, the greatest remedial agencies are air, light, food, exercise, sleep, and a peaceful, pure state of mind and heart.

That man is in the best health, then, who knows how, not most skillfully to compound medicines, but to wisely use these great natural health agencies which God has freely placed within the reach of us all.

In no department of hygiene is there so much room for the exercise of wisdom or folly as in our foods, and from nothing else do we actually receive such injury or benefit. From food, more than anything else, we must repair the wastes of the system and regulate its conditions.

Every substantial improvement, therefore, in the healthful preparation of food, is a boon which we may all welcome with gratitude. Now, no article occupies so conspicuous a place in our diet as wheat. This is a large part of every meal, and is justly called "the staff of life."

And yet, strange to say, this has never been prepared in a healthy way. The common white flour is robbed of a large part of the nerve, muscle, and bone nutriment of the wheat. To this we lay the blame of much of our nervousness, our universal loss of teeth, and considerable of our general ill-health,

Dr. Sylvester Graham saw very clearly this great deficiency of the white flour, and sought to remedy it by the introduction of what is called Graham flour. But in trying to remedy the difficulty he went too far. He not only gave us the whole wheat berry, which is all that is wanted, all that can be made a food, but he gave us the woody hull also, which has no nutrition in it, and which on the outside is covered with small sharp bristles, clearly seen under the microscope, that are very irritating to the delicate mucus membrane.

The deficient nutrition of the common white flour has been long well known by those who have given much attention to the study of health and food. The undesirableness of the hull of the Graham flour has also been seen and felt. This is the chief cause of the slow and hesitating manner with which it has been adopted.

But these two difficulties have at last been successfully overcome by the Health Food Company, 137 Eighth Street, New York. They have prepared wheat in may different forms—Pearled Wheat, Attrition Flour, Gluten, etc., all of them made from the best quality of wheat and very palatable and highly nutritious. From each of these preparations of wheat the hull is removed, and in the first two all parts of the wheat berry are retained. The attrition flour makes sweeter bread and is every way more healthy than the Graham flour, and must in time completely supersede it. To many stomachs the Gluten is especially adapted, because starch is hard to digest. The Gluten contains all the bone, muscle, and nerve-making food there is in the wheat. The writer can intelligently and heartily recommend it, especially to all persons of sedentary pursuits and constipated habits. He has tried it himself, and reaped the most satis factory results, and seen the most pleasing effects produced upon others by its use. Weak stomachs can easily dispose of it. Delicate membranes will not be irritated by it, and the bowels will soon be restored to their natural action. Thus the whole mucus membrane will by its use be soothed, healed, and invigorated. J. BASTOW.

PORT CHESTER, N. Y.

---

[From *Dr. Foote's Health Monthly* for October, 1876.]

A friend, who ought to know, says that the gluten of wheat is a splendid article of diet in cases of diabetes and in those of obesity. Our fat friends will be glad to learn the latter fact. The gluten of wheat can be obtained directly of the Health Food Company, 137 Eighth Street. We are also informed that the same article of diet is excellent in chronic dyspepsia.

---

[*From the* "*Living Issue*," *April* 27, 1876.]

WHOLE WHEAT FLOUR.

No whole wheat flour which we have ever examined equals the Cold-Air Attrition Flour, in the perfection of the pulverizing process. It is very clean, not dark-colored, because made from very choice, plump wheat; and the bread and cake made from it is sweet and delicious. It is not like "Graham," to be used *with* other bread foods, but it is to be used *instead* of all other breads. It is just the thing for mothers to bring up children upon. It is not merely a food for dyspeptics, but a food for well folks to keep well upon.

---

[*From* "*The Churchman*," *Sept.* 2, 1876.]

A SICK CLERGYMAN RESTORED.—The Rev. J. Bastow writes a letter to the Port Chester *Journal*, in which he says that the food-preparations of the Health Food Company, 137 Eighth Street, New York, have "satisfied strengthened, healed, and soothed me, as nothing has ever done since my health became impaired." He warmly recommends these foods to the sedentary, the dyspeptic, and the constipated.

---

[*From the* "*New York Eclectic Medical Journal*" *for Sept.* 1876.]

FOODS FOR THE SICK.

Perhaps we are not wholly just to our enterprising friends, the Health Food Company, of 137 Eighth Street, when we say that their chief work is to provide foods which can be appropriated by weak digestive machines. They are doing this very ably, as we personally know. But they are covering a much wider field than this. They are largely engaged in the manufacture of flour from wheat, by new and admirable methods, which conserve all that is useful and nourishing in the grain. This flour ought to be in every kitchen, and the bread made therefrom should be on every table in the land. We should expect to be called upon to treat very few cases of chronic dyspepsia and constipation if the use of this flour were universal.

Unquestionably there is sound philosophy in the theory so persistently urged by our friends, that the husky substance of the grains is bad for the stomach and bowels. We do need all that the hulls cover, but we do not need the hulls. Just below the hull is the best food of the cereal—the gluten—containing the mineral salts and the nitrogen. This part seems to emulsify with ease in stomachs so weak as to reject the most delicate gruels, paps and breads made from arrow-root or any starchy substance. We have had some very gratifying experiences in our own practice with the bland, soluble, nutritive foods prepared by the Health Food Company, and we are desirous that our friends of the profession should avail themselves of the advantages which thoughtful and earnest research in the domain of nutritive substances is competent to afford.

---

[*From the "Phrenological Journal" for September*, 1876.]

The Health Food Company, of No. 137 Eighth Street, New York, whose advertisement will be found in another column, have a theory with reference to the preparations of wheat they offer that does not tally exactly with the views we have been advocating for many years ; but, as they are making commendable efforts to induce people to give over the use of fine flour and other things which we know to be injurious, we have no objection to state briefly, for the information of our readers, the grounds on which they claim superiority for their productions.

They claim that many stomachs will not perfectly assimilate foods made from crushed or cracked wheat or Graham flour, as generally sold. The hully matter, they assert, is not dissolved either by cooking or by the power of the gastric fluids, and the food accordingly ferments instead of emulsifying in the stomach, and induces distension by the gases evolved, as well as acidity and discomfort. The scratching power of the hulls induces an outpouring of fluid along the intestinal tract to protect the mucus membrane from abrasion. Tne effect of the daily use of food containing the silicious flakes of wheat bran which are, of course, insoluble, they claim to be similar to catharsis or purgation from any other cause. This double source of injury, malnutrition, by reason of the failure of the process of digestion in the stomach owing to the insolubility of its contents and the depletion of the system by the drain made upon it for lubricating material against irritants in the intestines, they allege is shown to have been the cause of the discomfort and thinness of body from which many intelligent people have suffered, when seeking to live hygienically. They therefore strip the woody hulls from the grains before cracking or pulverizing, leaving all the gluten and other food elements. In this way they prepare Graham flour and cracked or coarse granulated wheat, which is said to readily digest in even delicate stomachs.

We are well acquainted with the Health Food Company, who are our neighbors, and can commend them for integrity and enthusiastic devotion to their business. Our readers who may desire to learn more concerning their views of what constitutes a proper diet, can obtain much interesting reading matter by writing to them and stating that this notice was seen in the *Phrenological Journal.*

